# Neural Mechanisms Underlying Breast Cancer Related Fatigue: A Systematic Review of Neuroimaging Studies

**DOI:** 10.3389/fnins.2021.735945

**Published:** 2021-11-10

**Authors:** Nisha Arya, Anya Vaish, Ke Zhao, Hengyi Rao

**Affiliations:** Department of Neurology, Center for Functional Neuroimaging, University of Pennsylvania Perelman School of Medicine, Philadelphia, PA, United States

**Keywords:** brain network, breast cancer, cancer related fatigue, connectivity, fMRI

## Abstract

**Introduction:** Fatigue and cognitive dysfunction commonly co-occur in breast cancer patients and survivors. However, the underlying neural mechanism is not clear. We performed a systematic review of studies that used neuroimaging methods to investigate structural and functional changes in the brain associated with fatigue in breast cancer patients and survivors.

**Methods:** We searched PubMed, Scopus, EmBase, and Cochrane CENTRAL from January 2009 to May 2021 for studies that reported brain neuroimaging findings in relationship to fatigue in breast cancer patients or survivors. Neuroimaging methods included magnetic resonance imaging (MRI), positron emission tomography (PET), and electroencephalogram (EEG). We summarized structural and functional neuroimaging changes associated with fatigue.

**Results:** Of the 176 articles retrieved, ten MRI studies reported neuroimaging findings in relationship to fatigue. Together these studies compared 385 breast cancer patients or survivors to 205 controls. Fatigue was associated with reduced white matter integrity and increased glutamate in the insula but changes in gray matter volume were not associated with fatigue score. Nine of the ten studies found significant associations between fatigue and functional changes in the frontoparietal cortex. In response to memory and planning tasks, fatigue was associated with increased activations in several regions of the frontoparietal cortex, however, overall performance on tasks was not reduced. Fatigue was also associated with extensive changes in the connectivity of brain networks that filter endogenous signals (salience network), internal attention (default mode network), and external attention (dorsal attention network). Subcortical regions associated with fatigue included insula (interoception), superior colliculus (sleep regulation), and thalamus (alertness). Functional brain changes before initiation of chemotherapy were a better predictor of post-treatment fatigue than chemotherapy itself.

**Conclusions:** Fatigue in breast cancer is associated with widespread functional changes of brain regions and networks that affect executive function including memory, planning, internal and external attention. Observed changes likely represent a compensatory mechanism through which breast cancer patients and survivors try to maintain adequate executive function. Breast cancer patients scheduled to undergo chemotherapy are at high risk for developing fatigue even before the start of treatment.

## Introduction

Fatigue is the most common and distressing side effect of breast cancer (BC) treatment (Berger et al., 2012). Cancer-related fatigue (CRF) is defined as a distressing, persistent, subjective sense of physical, emotional, and/or cognitive tiredness or exhaustion related to cancer or cancer treatment that is not proportional to recent activity and interferes with usual functioning (Berger et al., 2015). Depending on the diagnostic criteria used, the prevalence of CRF ranges from 56 to 95% with some BC survivors reporting persistent fatigue years after completion of cancer treatment (Bower et al., 2006). CRF has a severe impact on quality of life by disrupting participation in daily vocational and social activities (Bower et al., 2006). This has led the National Cancer Institute to identify CRF as one of the top five first-tier high-priority research areas (National Institutes of Health State of the Science Panel, 2003).

BC patients with CRF commonly report both physical and mental fatigue. Physical fatigue specifically refers to physical sensations related to fatigue. Mental fatigue pertains to a variety of distressing cognitive symptoms including diminished concentration and attention, difficulty completing daily tasks, and perceived problems with short-term memory (Lin et al., 2009). This cluster of symptoms, which is often observed during chemotherapy, is colloquially referred to as “chemo brain” (Askren et al., 2014). Several studies have reported that patients with BC report fatigue and demonstrate worse cognitive performance on neuropsychological assessments even before the start of chemotherapy (Wefel and Schagen, 2012; Lange et al., 2014). These findings suggest that a central neurobiological mechanism likely contributes to CRF; however, the underlying neural mechanism is not known.

Functional magnetic resonance imaging (fMRI) methods have allowed researchers to non-invasively study brain structure and function. Blood oxygen level dependent (BOLD) signals can be used to measure effects of fatiguing cognitive tasks on brain function and identify regions of interest in the brain associated with CRF. Similarly, fMRI in the resting state can be used to measure how CRF affects functional connectivity between regions and networks of interest. Finally, magnetic resonance spectroscopy and diffusion tensor imaging can be used to non-invasively determine the association of fatigue with brain metabolites and structural changes in white matter, respectively. Additional neuroimaging methods to investigate functional brain changes include positron emission tomography (PET scan) and electroencephalography (EEG). PET activation studies allow measurement of cerebral activation during fatiguing tasks while fluorodeoxyglucose (FDG)-PET scanning allows assessment of brain metabolism. EEG allows measurement of changes in brain electrical activity and how these changes correlate with fatiguing cognitive and physical tasks. A prior systematic review has documented brain changes following chemotherapy for BC (Pomykala et al., 2013a). These changes include reductions in gray matter volume and decreases in white matter integrity, as well as task-related hypo-and hyper activations and changes in brain metabolism and electrical activity in several regions months to years following therapy. However, neuroimaging findings in relationship to CRF were not reported. Several studies have investigated the relationship of CRF with structural and functional changes in the brain in BC patients or survivors, yet a systematic review that integrates these findings is lacking. Neuroimaging changes associated with CRF could not only elucidate central neural mechanisms underlying CRF, but also help clinicians understand the extent to which chemotherapy or other BC treatments contribute to CRF. Finally, neuroimaging findings could allow the development of pre-treatment biomarkers for identifying BC subjects at high risk for developing CRF.

We performed a systematic review of studies that used neuroimaging including functional MRI, EEG, and PET scan to investigate structural and functional changes in the brain associated with CRF. Our aim was to understand the neural mechanisms underlying CRF in subjects who are diagnosed with or currently undergoing treatment for BC (BC patients) and patients who have completed treatment for BC (BC survivors).

## Methods

We reviewed published studies that reported neuroimaging findings in relationship to fatigue in BC patients or survivors. The study was conducted using detailed methodological guidance for systematic review guidelines (PRISMA statement), (Moher et al., 2009). Participants consisted of BC patients (currently undergoing treatment for breast cancer) or BC survivors (completed treatment for breast cancer). The comparison groups consisted of subjects without fatigue (either healthy controls or BC subjects/survivors without fatigue). Our primary outcome was brain region activated in relationship to fatigue. Secondary outcomes were connectivity changes, structural changes, and changes in brain metabolite in relationship to fatigue. Our clinical outcome was fatigue score as measured by a validated questionnaire.

Neuroimaging methods included functional magnetic resonance imaging, positron emission tomography (PET scan) and electroencephalography (EEG). Functional MRI studies included diffusion tensor imaging for white matter integrity, magnetic resonance spectroscopy for brain metabolites, blood oxygen level dependent functional magnetic resonance imaging (BOLD fMRI), and resting state functional connectivity magnetic resonance imaging.

### Eligibility Criteria

Inclusion and exclusion criteria, search strategy including methods for assessing risk of bias, and outcomes to be measured were specified in advance and documented in a protocol. We included studies that:

1. Imaged female subjects who were currently undergoing treatment for BC (BC patients) or who had completed treatment for BC (BC survivors)
2. Measured fatigue using a validated questionnaire
3. Reported neuroimaging findings in relationship to fatigue
4. Used observational study designs (cross-sectional, prospective, or case-control studies) and interventional study designs (randomized controlled trials).

We excluded studies that (1) included patients with cancers other than BC; (2) reported clinical neuroimaging findings for indications such as brain metastasis; and (3) were abstracts only, case reports, reviews, and articles not published in English.

### Search Method

We performed a comprehensive search of the following databases from years 1990 to May 25, 2020: PubMed, Scopus, and Embase, and Cochrane Central Register for Clinical Trials To identify functional MRI studies, we combined MeSH terms “brain” AND “MRI” AND “breast cancer” AND “fatigue.” PET scan studies were identified by combining MeSH terms “brain” AND “PET” AND “breast cancer” AND “fatigue.” EEG studies were identified by combining MeSH terms “brain” AND “EEG” AND “breast cancer” AND “fatigue.” We also performed a manual search of bibliographies of the identified papers to find additional studies. The title and abstract of all studies retrieved through the search strategy were first screened using Endnote, and duplicates were removed. Two reviewers also performed a critical appraisal of the remaining studies using a checklist to determine if they met our inclusion and exclusion criteria.

### Risk of Bias

Risk of bias was assessed by (1) comparing age of the patients in the study and comparison groups; (2) determining if fatigue scores on a validated questionnaire were reported; (3) assessing if studies measured at least one covariate (such as anxiety, depression, worry) that could confound the relationship between CRF and neuroimaging findings. We specified *a priori* in our protocol that risk of bias would be considered low if all three criteria were met, moderate if one or two criteria were met, and high if no criteria were met. Each study was reviewed for bias by two reviewers independently and disagreements between the two were resolved by the senior author.

### Data Extraction

The following data was extracted using structured forms: (1) publishing data (author, year); (2) study design (cross-sectional, prospective, or randomized clinical trial) including covariates measured; (3) participants: number of subjects in study and comparison groups, age, BC treatment status (before treatment, currently undergoing or immediately after treatment, survivor); (4) neuroimaging techniques including strength of scanner, activation tasks, and methodology of neuroimaging technique; (5) clinical outcome: the name of the questionnaire used to measure fatigue, fatigue scores in the study group and the comparison group; (6) neuroimaging results: region of interest, connectivity findings, brain metabolite, structural changes. Data was extracted independently by two authors and consistency between the two was assessed.

### Data Analysis

First, bibliometric analysis was used to analyze characteristics of included studies. Risk of bias in included studies was assessed. Then we narratively described the neuroimaging results in terms of their relationship with CRF. We did not perform a quantitative meta-analysis of neuroimaging data because of heterogeneity in the neuroimaging methods of included studies and because all studies did not report xyz coordinates.

## Results

### Studies

The three searches together yielded 176 articles.

**Figure 1:**
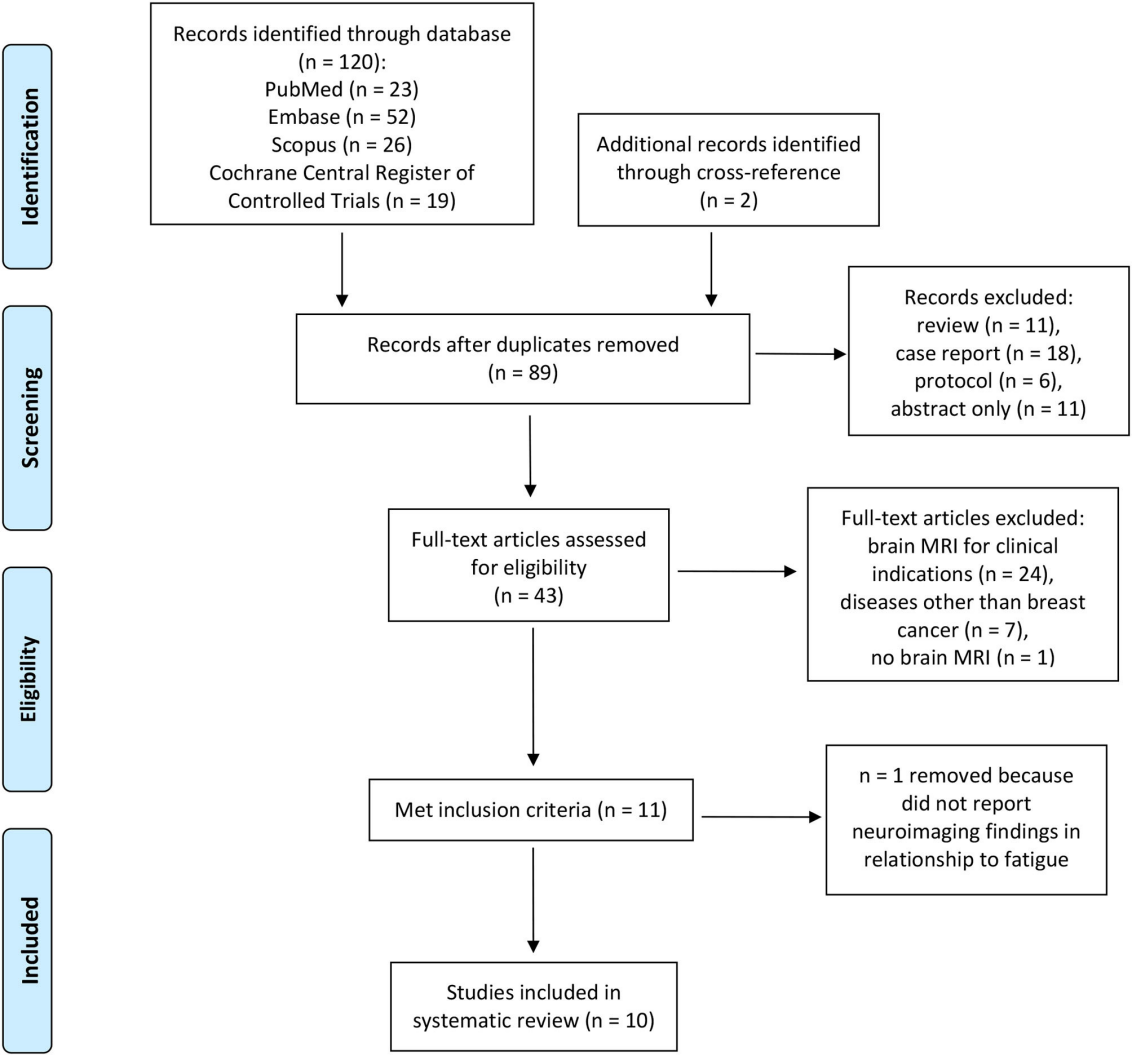
PRISMA flow diagram of functional MRI studies on breast cancer related fatigue (10).

The database search for MRI studies resulted in 120 studies with two additional studies identified through cross-references. The PRISMA flow diagram showing the selection process for MRI studies is shown in Figure 1. After removing duplicates, reviews, case reports, protocols, and abstracts only, a total of 43 articles were assessed for eligibility. One study that reported neuroimaging findings in BC patients was removed because findings in relationship to fatigue were not reported (Vardy et al., 2019). Another paper that investigated structural changes and brain metabolites in relationship to CRF in patients with Hodgkin's lymphoma was also removed (Prinsen et al., 2013).

The search for PET scan identified 34 papers. None of the papers met the eligibility criteria of our systematic review. Detailed reasons for excluding these papers are as follows: 12 were duplicates, two were case reports, two were reviews, two included patients with cancers other than BC, 11 reported clinical PET scan findings for indications such as brain metastasis, and five did not report PET scan findings in relationship to fatigue.

This search for EEG identified 20 papers. None of the papers met the eligibility criteria of our systematic review. Detailed reasons for excluding these papers are as follows: eight were duplicates, two were case reports, one was a review, five included patients with cancers other than BC, two were abstract-only, and two did not report EEG findings in relationship to fatigue.

The 10 studies that met our inclusion and exclusion criteria are listed in Table 1. All of these studies had used functional MRI as the neuroimaging method. Of these, 40% (4/10) were cross-sectional in design (Zick et al., 2014; Churchill et al., 2015; Hampson et al., 2015; Menning et al., 2015), 40% (4/10) were prospective (McDonald et al., 2012; López Zunini et al., 2013; Askren et al., 2014; Menning et al., 2017), and 20% (2/10) were randomized clinical trials (Harris et al., 2017; Van der Gucht et al., 2020). The two randomized controlled trials compared the effect of interventions: relaxing vs. stimulating acupressure (Harris et al., 2017), and mindfulness vs. waitlist control condition in fatigued BC survivors (Van der Gucht et al., 2020).

**Table 1:** Neuroimaging studies that used MRI to evaluate neural mechanisms of breast cancer related fatigue.

**Author, year**	**Study design**	**Population**	**Imaging technique,** **scanner**	**Fatigue questionnaire**	Covariates^*^
Zick et al. (2014)	Cross-sectional	16 fatigued BC survivors, 13 non-fatigued BC survivors	Brain biochemistry using MRS, 3T	Average brief fatigue inventory score ≥ 4.0	Anxiety, sleep, pain
Menning et al. (2015)	Cross-sectional	32 BC patients scheduled for chemotherapy, 33 BC patients not scheduled for chemotherapy, 38 no-cancer controls	fMRI planning task, fMRI memory task, DTI for white matter integrity, MRS for brain metabolites 3T	Profile of mood states and European organization for research and treatment of cancer quality of life questionnaire	Anxiety, depression, stress, mood
Churchill et al. (2015)	Cross-sectional	65 BC patients scheduled for CT/radiation, 32 healthy controls	fMRI verbal working memory task 3T	Functional assessment of chronic illness therapy-fatigue	Anxiety, depression, worry, sleep
Hampson et al. (2015)	Cross-sectional	15 fatigued BC survivors, 8 non-fatigued BC survivors	Resting state connectivity using fcMRI, 3T	Average brief fatigue inventory score ≥ 4.0	Anxiety, depression, sleep, pain
McDonald et al. (2012)	Prospective (baseline, 1 month, and 1 year after treatment)	16 BC patients treated with chemotherapy, 12 BC patients not treated with chemotherapy, 15 healthy controls	fMRI working memory, 1.5T	Fatigue symptom inventory	Anxiety, depression
López Zunini et al. (2013)	Prospective (baseline and 1 month after chemotherapy)	21 BC patients before and after chemotherapy, 21 healthy controls	fMRI verbal recall task, 1.5T	Profile of mood states	Anxiety, depression, anger
Askren et al. (2014)	Prospective (baseline and 1 month after chemotherapy)	28 BC patients treated with chemotherapy, 37 BC patients not treated with chemotherapy, 32 healthy controls	fMRI verbal working memory task, 3T	Functional assessment of chronic illness therapy-fatigue	Worry
Menning et al. (2017)	Prospective study (baseline and 6 months after treatment)	28 BC patients scheduled to receive systemic treatment, 24 BC patients not scheduled for systemic treatment, 31 no-cancer controls	fMRI planning task, fMRI memory task, 3T	Profile of mood states and European organization for research and treatment of cancer quality of life questionnaire	Anxiety, depression, mood
Harris et al. (2017)	Randomized controlled trial	9 fatigued BC survivors scheduled for relaxing acupressure, 10 fatigued BC survivors scheduled for stimulating acupressure	Resting state connectivity using fcMRI, brain metabolites using Proton MRS, 3T	Average brief fatigue inventory score ≥ 4.0	Sleep, subjects with depression excluded
Van der Gucht et al. (2020)	Randomized controlled trial	18 BC survivors scheduled for mindfulness intervention, 15 BC survivors on waitlist control condition	fMRI and fcMRI before and after intervention, 3T	Fatigue severity subscale of checklist individual strength (fatigue score > 35)	Depression

*BC, breast cancer; fMRI, functional magnetic resonance imaging; fcMRI, functional connectivity magnetic resonance imaging; 1.5 or 3T, Strength of Scanner Magnet in Tesla Unit; MRS, proton magnetic resonance spectroscopy*.

* *Potential confounders of the association between cancer related fatigue and neuroimaging findings*.

### Risk of Bias

No significant difference was noted in the age of the participants of the study and comparison groups in nine of the 10 studies included in our analysis (Table 2). All studies measured fatigue using a validated questionnaire and all studies measured at least one covariate that was a potential confounder of fatigue (Table 1). The most common covariates measured were anxiety and depression (each measured by 80% of the studies), followed by sleep (40%), worry (20%), and pain (20%), (Table 1). Of the 10 studies, the majority (70%) measured three or more confounders, one study (10%) measured two confounders, and two studies (20%) measured one confounder. The overall risk of bias was considered low for all studies except one by Churchill et al. (2015). That study was considered to have moderate risk of bias because age and fatigue scores were not reported, though fatigue as well as several covariates (anxiety, depression, worry, and sleep) were measured using validated questionnaires (Churchill et al., 2015).

**Table 2:** Population of breast cancer patients and survivors investigated for neural mechanisms of breast cancer related fatigue.

**Author, year**	**Study group**	**Age**	**Fatigue score^1^**	**Comparator group**	**Age**	**Fatigue score**
McDonald et al. (2012)	16 BC patients (chemotherapy+) 12 BC patients (chemotherapy–)	53 ± 9 53 ± 7	3.2 ± 2 3.2 ± 1.7	15 healthy controls	50 ± 6	3.3 ± 1.8
López Zunini et al. (2013)	21 BC patients (before and after chemotherapy)	50.6 ± 8.3	7.4 ± 5.8^a^^,^^b^	21 healthy controls	49.7 ± 8.8	4.4 ± 3.4
Askren et al. (2014)	28 BC patients (chemotherapy+) 37 BC patients (chemotherapy– and radiation +)	50 ± 10 53 ± 9	13.4 ± 10.9^a^^,^^b^ 9.6 ± 7.7	32 healthy controls	50 ± 9	8.1 ± 7.8
Zick et al. (2014)	16 fatigued BC survivors	57 ± 8	4.8 ± 1.2 ^a^	13 non-fatigued BC survivors	57 ± 9	1.5 ± 1.2
Menning et al. (2015)	32 BC patients (chemotherapy+) 33 BC patients (chemotherapy–)	50 ± 9 52 ± 7	24 ± 23^a^ 34 ± 25^a^	38 no-cancer controls	50 ± 9	15 ± 19
Churchill et al. (2015)	28 BC patients (chemotherapy+) 37 BC patients (radiation+)	NR	NR	32 healthy controls	NR	NR
Hampson et al. (2015)	15 fatigued BC survivors	57 ± 9	15.8 ± 4^a^	8 non-fatigued BC survivors	55 ± 8	11.8 ± 4
Harris et al. (2017)	9 fatigued BC survivors (relaxing acupressure+)	59 ± 6	−1.8 ± 1.5^d^	10 fatigued BC survivors (stimulating acupressure +)	60 ± 9	−1.8 ± 1.5
Menning et al. (2017)	28 BC patients (systemic treatment+) 24 BC patients (systemic treatment–)	49 ± 9 51 ± 7	32 ± 26^a^ 19 ± 22	31 no-cancer controls	51 ± 8	13 ± 15
Van der Gucht et al. (2020)	18 BC survivors (mindfulness intervention+)	44 ± 6	34.7 ± 8	15 BC survivors (mindfulness intervention –)	47 ± 5	39.3 ± 8
**Total:**	385			205		

*(chemotherapy+) or (chemotherapy–), group received or did not receive chemotherapy; (systemic treatment+) or (systemic treatment–), group received or did not receive systemic treatment*.

1 *Fatigue Score, For all Instruments, Higher Score Indicates Worse Symptoms*.

a *P-value significantly higher as compared to control group*.

b *P-value at baseline was significant lower than after chemotherapy*.

d *significant improvement in fatigue score with treatment. NR, Not reported*.

### Participants

Together, the 10 studies compared functional MRI neuroimaging findings in 385 BC patients or survivors to 205 controls. In one study, neuroimaging was performed prior to the start of chemotherapy (Churchill et al., 2015). In 50% (5/10) of studies, neuroimaging was performed before and after completion of chemotherapy (McDonald et al., 2012; López Zunini et al., 2013; Askren et al., 2014; Menning et al., 2015, 2017). In 40% (4/10) studies, neuroimaging was performed in fatigued BC survivors (Zick et al., 2014; Hampson et al., 2015; Harris et al., 2017; Van der Gucht et al., 2020), (Table 2). The comparison group consisted of no-cancer controls in 60% of the studies (McDonald et al., 2012; López Zunini et al., 2013; Askren et al., 2014; Churchill et al., 2015; Menning et al., 2015, 2017), 20% studies compared fatigued BC survivors to non-fatigued BC survivors (Zick et al., 2014; Hampson et al., 2015) and 20% were interventional studies in fatigued BC survivors (Table 2). In one interventional study, fatigued BC survivors undergoing relaxing acupressure were compared to fatigued BC survivors undergoing stimulating acupressure (Harris et al., 2017). In the other interventional study, BC survivors undergoing mindfulness meditation were compared to BC survivors on a control waitlist (Van der Gucht et al., 2020).

### Neuroimaging Methods and Outcomes

#### MRI Scanner

The majority of the studies (8/10) were performed on higher resolution 3T MRI scanners (Askren et al., 2014; Zick et al., 2014; Churchill et al., 2015; Hampson et al., 2015; Menning et al., 2015, 2017; Harris et al., 2017; Van der Gucht et al., 2020), (Table 1). Only two studies were performed on 1.5T scanners (McDonald et al., 2012; López Zunini et al., 2013).

#### Activation Task

Six studies used BOLD fMRI to measure brain activation in response to a fatiguing cognitive task, either a verbal memory or a planning task or both (McDonald et al., 2012; López Zunini et al., 2013; Askren et al., 2014; Churchill et al., 2015; Menning et al., 2015, 2017), (Table 3). The verbal task used in these studies is known to activate the frontoparietal regions (Owen et al., 2005). Of the six studies that used a verbal task, one study used a relatively easy verbal recall task (López Zunini et al., 2013), three studies used a more complex verbal working memory task (McDonald et al., 2012; Askren et al., 2014; Churchill et al., 2015), and two studies used the paired associates verbal memory task which has high task difficulty and been shown to reliably activate the parahippocampal region (Jager et al., 2007; Menning et al., 2015, 2017). Two studies also used an additional visuospatial planning task using the Tower of London paradigm to test executive function (Menning et al., 2015, 2017), (Table 3).

**Table 3:** Brain regions and networks associated with breast cancer related fatigue.

**Author, year**	**Neuroimaging state**	**Regions of interest/network**	**Region/network associated with CRF?**	**xyz coordinates^a^**
**TASK-RELATED ACTIVATIONS**				
McDonald et al. (2012)	Verbal working memory *n*-back task	Increased activation of frontoparietal regions but no region associated with CRF	No	Not reported
López Zunini et al. (2013)	Verbal recall memory task	**Increased activation** Right medial frontal gyrus Left hippocampus **Reduced activation** Right anterior cingulate	Yes	10 36 46 −32 −28 −8 4 10 26
Askren et al. (2014)	Verbal working memory task	**Reduced neural efficiency** Frontoparietal executive network	Yes	Marker of neural efficiency associated with CRF
Menning et al. (2015)	Planning task Paired associates verbal memory task	**Increased activation** Dorsomedial pre-frontal cortex No changes	Yes	36 16 52 32 26 50
Churchill et al. (2015)	Verbal working memory task	Inferior and superior frontal lobe Dorsolateral pre-frontal cortex Parietal region	Yes	H factor (marker of neuronal processes) associated with CRF
Menning et al. (2017)	Planning task Paired associates verbal memory task	**Increased activation** Right inferior parietal cortex No changes	Yes	36 −40 38 30 −42 38 34 −40 50
**CONNECTIVITY CHANGES**				
Hampson et al. (2015)	Resting state	**Fatigue** **>** **non-fatigue** Inferior parietal lobule to superior frontal gyrus (physical fatigue) DMN to superior frontal gyrus (mental fatigue) Medial frontal gyrus to inferior parietal lobule (mental fatigue) Posterior cingulate to cerebellum **Non-fatigue** **>** **fatigue** Right precuneus to the superior colliculus/periaqueductal gray Inferior parietal lobule to right parahippocampus Left inferior parietal lobule to subgenual anterior cingulate Left posterior cingulate to middle temporal gyrus Left precuneus to primary somatosensory	Yes	34 28 48 −35 31 37 44 −68 34 52 −72 −40 2 −34 −4 26 −36 −12 −4 16 −26 40 −54 4 −48 −18 42
Harris et al. (2017)	Resting state	Insula to dorsolateral pre-frontal cortex DMN to left pulvinar thalamus DMN to right pulvinar thalamus DMN to superior colliculus/periaqueductal gray	Yes	−37 45 21 −19 −31 15 13 −33 13 −3 −31 −3
Van der Gucht et al. (2020)	Resting state	Between dorsal and salience attention networks	Yes	Not reported

a *xyz Coordinates of Peak Voxel of Region Associated With CRF Using MNI (Montreal Neurological Institute) System. DMN, Default mode network*.

#### Outcomes

The majority (60%) of studies reported regions of interest or network activated in response to memory or planning task (Table 3), (McDonald et al., 2012; López Zunini et al., 2013; Askren et al., 2014; Churchill et al., 2015; Menning et al., 2015, 2017). Askren et al. (2014) measured neural efficiency which is a marker of energy consumption of a brain region in response to a given task. In this study, spatial variance of the magnitude of fMRI activations averaged across all of the voxels of the frontoparietal executive network was used as a marker of neural efficiency. Churchill et al. (2015) used the Hurst component, a quantitative measure of neuronal processes involved in working memory, to identify regions of interest associated with CRF.

Three studies reported changes in resting state connectivity using fcMRI (Hampson et al., 2015; Harris et al., 2017; Van der Gucht et al., 2020). Two studies also performed magnetic resonance spectroscopy for brain metabolites (Menning et al., 2015; Harris et al., 2017) and one study reported white matter changes on diffusive tensor imaging (Menning et al., 2015).

### Clinical Outcome

All studies measured fatigue using a validated questionnaire (Table 1). Fatigue scores as measured by validated questionnaires for the study and comparison groups are shown in Table 2. Fatigue scores in BC patients were higher (worse) at baseline even before the start of chemotherapy as compared to healthy controls in four studies (López Zunini et al., 2013; Askren et al., 2014; Menning et al., 2015, 2017). Fatigue scores were also higher in BC patients awaiting chemotherapy than BC patients not scheduled for chemotherapy or scheduled for other treatments such as radiation (Askren et al., 2014; Menning et al., 2015, 2017). In three prospective studies, fatigue scores were significantly higher (worse) after treatment than at baseline (López Zunini et al., 2013; Askren et al., 2014; Menning et al., 2017).

In the two interventional studies investigating the effect of acupressure and mindfulness on CRF, fatigue scores significantly improved with treatment (Harris et al., 2017; Van der Gucht et al., 2020).

In all studies, the relationship of CRF with observed brain changes was reported after controlling for the confounding effect of at least one psychological covariate (such as anxiety, worry, or depression).

### Brain Regions Associated With CRF in BC

**Figure 2:**
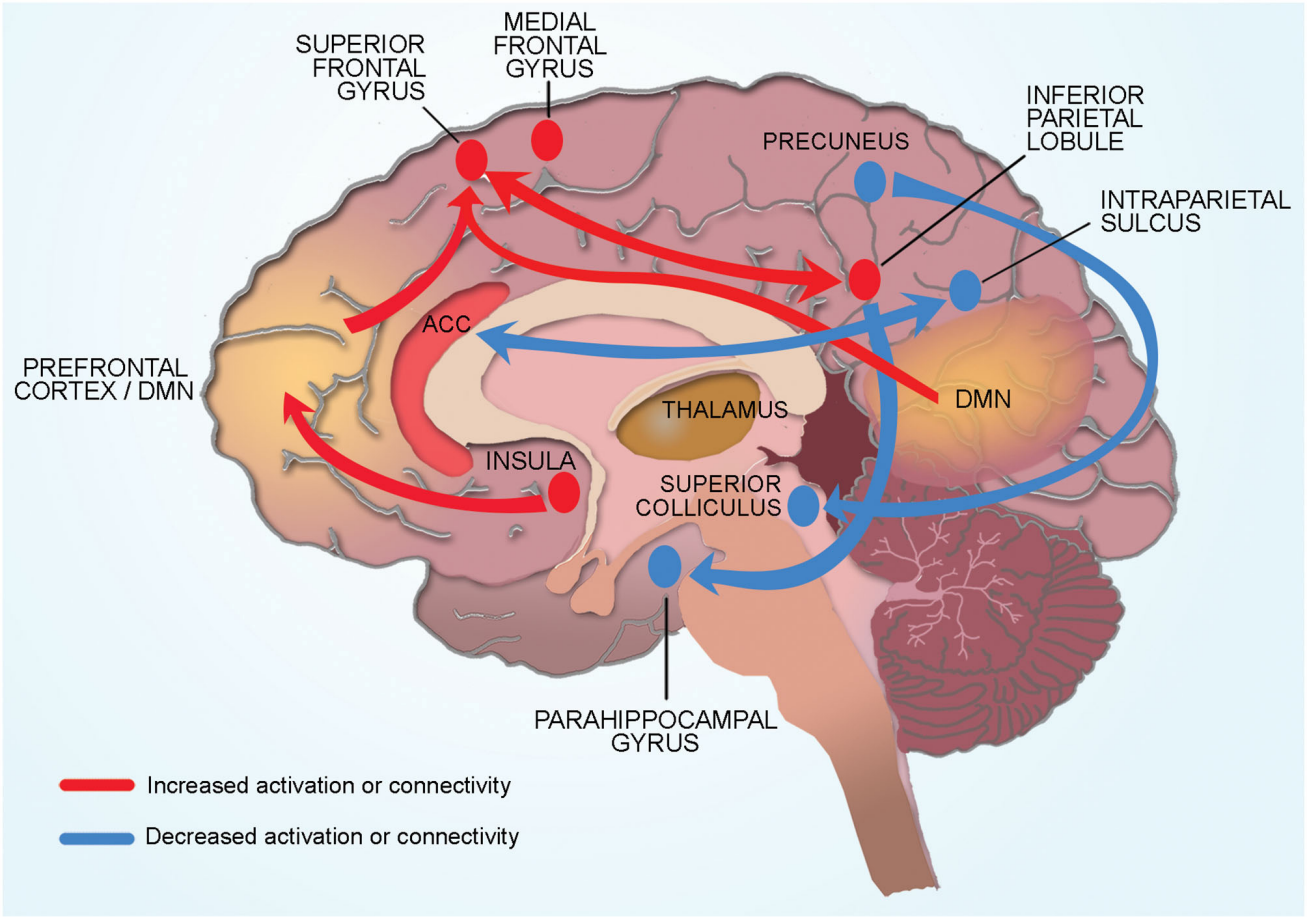
Conceptual model of neural mechanisms of CRF. Cancer related fatigue is associated with compensatory hyper activation of multiple cortical regions involved in memory and planning (dorsomedial pre-frontal cortex, medial frontal gyrus, inferior parietal cortex). Connectivity changes in cortical and subcortical regions also contribute to CRF. Physical fatigue is associated with increased connectivity between the left inferior parietal lobule and superior frontal gyrus (region associated with memory and cognition). Increased connectivity of the default mode network to the superior frontal gyrus increases self-referential thoughts and a sensation of mental fatigue. Increased connectivity of the insula (processes internal body signals) to the dorsolateral pre-frontal cortex may lead to greater self-referential appraisal of fatigue. Reduced connectivity between the salience network (anterior cingulate cortex) and the dorsal attention network (intraparietal sulcus) contribute to CRF through hypervigilance of internal bodily signals and reduced engagement with the external environment. Subcortical structures such as superior colliculus and pulvinar (thalamus) contribute indirectly to CRF by affecting sleep and alertness. Connectivity of the right precuneus to the superior colliculus (sleep regulation) and of the inferior parietal lobule to parahippocampal gyrus (memory encoding and retrieval) may be protective against the development of CRF.

Even before initiation of treatment, CRF was associated with increased task-related activation in multiple regions of the frontoparietal cortex that support working memory and attention including the dorsomedial pre-frontal cortex, medial frontal gyrus, and inferior parietal cortex (Table 3; Figure 2). However, these hyper activations were not associated with any significant reduction in task performance (McDonald et al., 2012; López Zunini et al., 2013; Menning et al., 2015, 2017).

Several studies reported that anxiety and/or depression modulated the association between CRF and frontoparietal activations (McDonald et al., 2012; López Zunini et al., 2013; Askren et al., 2014; Churchill et al., 2015; Menning et al., 2015). López Zunini et al. (2013) reported that observed associations between CRF and brain activations following chemotherapy disappeared after adjusting for pre-chemotherapy levels of fatigue and anxiety scores indicating that baseline fatigue and anxiety are more important contributors to CRF than chemotherapy. Using spatial variance, a marker of neural efficiency, Askren et al. (2014) demonstrated that pre-treatment reduced neural efficiency of the frontoparietal network that supports executive function was a better predictor of post-chemotherapy fatigue than chemotherapy itself.

The type of treatment also had a significant impact on fatigue scores and brain activations even before initiation of treatment. Specifically, planned chemotherapy was associated with worse fatigue scores than planned radiation or no additional treatment (Table 3). BC patients scheduled to undergo chemotherapy showed worse neural efficiency in the frontoparietal executive network even before initiation of chemotherapy than patients awaiting radiotherapy (Askren et al., 2014). Chemotherapy was also associated with greater task-related brain activations than radiation or no additional treatment. McDonald et al. (2012) demonstrated that BC patients who received chemotherapy demonstrated greater frontal activations 1 year after chemotherapy compared to BC patients who did not receive chemotherapy. Similarly, Menning et al. (2017) demonstrated that with increasing executive functioning task load, BC patients who received systemic treatment (with chemotherapy ± hormones) showed increased parietal activation compared to baseline compared to BC patients who did not receive systemic treatment.

Only the study by López Zunini et al. (2013) reported that CRF was associated with reduced task related activation of the anterior cingulate cortex.

Only one study was unable to identify any task-related activation associated with CRF. McDonald et al. (2012) reported greater frontal and parietal activation during working memory performance in BC patients as compared to controls, however, no association with CRF was seen. The authors noted that this was likely because fatigue scores were high in the control group resulting in no significant difference in fatigue scores between BC patients and no cancer controls. Two studies did not find a relationship between CRF and a memory task likely because the paired associates memory task was too difficult, reflected in a floor effect in behavioral as well as imaging data (Menning et al., 2015, 2017).

### Brain Networks Associated With CRF in BC

Resting state connectivity studies in BC survivors showed that CRF was associated with changes in connectivity between several regions of the frontoparietal cortex, many of which are near regions identified in task-activation studies. Specifically, physical fatigue was associated with increased connectivity between the left inferior parietal lobule and superior frontal gyrus while mental fatigue was associated with increased connectivity of the medial frontal gyrus to inferior parietal lobule.

CRF was associated with abnormal connectivity of the default mode network (DMN), (Table 3; Figure 2). Specifically, mental fatigue was associated with greater connectivity between the DMN and the superior frontal gyrus (involved with memory and cognition) in fatigued BC survivors (Table 3; Figure 2). The opposite relationship was noted in non-fatigued survivors such that reduced connectivity between the DMN and superior frontal gyrus was associated with less fatigue. Similarly, connectivity between the right precuneus and superior colliculus (associated with sleep regulation) was higher in non-fatigued than fatigued BC survivors indicating that some connectivities may serve a protective function by inhibiting fatigue.

Two randomized controlled trials reported that improvement in fatigue scores with interventions was associated with changes in brain connectivity patterns between brain networks and subcortical structures (Table 3; Figure 2). In a randomized controlled trial comparing stimulating vs. relaxing acupressure in fatigued BC survivors, Harris et al. (2017) reported that both kinds of acupressure improved fatigue and sleep scores. This improvement was associated with changes in connectivity of the insula (processes internal body signals) to the dorsolateral pre-frontal cortex (involved in “top down” regulation of fatigue), DMN to the superior colliculus/periaqueductal gray (sleep regulation), and DMN to the pulvinar/thalamus (alertness). Similarly, Van der Gucht et al. (2020) reported that mindfulness intervention improved in fatigue scores and increased connectivity between the salience network (filtering endogenous signals) and dorsal attention network (involved with external attention). In these studies, a direct relationship between change in fatigue scores and change in connectivity was not observed likely due to small sample size.

#### CRF and Brain Metabolites

Two studies investigated the relationship between brain metabolites measured using magnetic resonance spectroscopy and peripheral inflammatory biomarkers in blood and hair samples (Zick et al., 2014; Menning et al., 2015). Fatigued BC survivors had significantly higher ratios of the brain metabolite markers, glutamate + glutamine (Glx) to N-acetyl-aspartate (NAA), (*p* = 0.01), and creatine to total creatine (Cr/tCr), (*p* = 0.03) in the posterior insula compared to non-fatigued BC survivors. Serum IL-6 was increased in fatigued women compared to non-fatigued women (*p* = 0.03); however, posterior insula Glx/NAA, Cr/tCr and serum IL-6 were not significantly correlated with one another. Menning et al. (2015) did not find differences in brain metabolites in left semioval center and left hippocampus between BC patients and no-cancer controls; hair cortisol (marker of stress) was also not different between groups.

#### CRF and Structural Changes in the Brain

Only one study investigated structural changes in the brain in BC patients in relationship to CRF. Using diffusion tensor imaging of the entire brain, Menning et al. (2015) reported that in patients diagnosed with BC but who had not yet initiated treatment demonstrated lower white matter integrity as indicated by lower fractional anisotropy and higher mean diffusivity as compared to healthy controls. These differences were no longer significant when fatigue was accounted for indicating that CRF may be associated with white matter changes. CRF was not associated with changes in gray matter brain volume of dorsolateral pre-frontal cortex and the superior parietal cortex.

## Discussion

A review of findings of multi-modal brain imaging methods reveals that central neural mechanisms are an important contributor to CRF. CRF in BC patients and survivors is associated with few structural but extensive functional changes in cortical and subcortical regions and networks that process working memory, interoception, internal and external attention. Though it is unclear if the observed changes are the cause or the effect of CRF, they provide a neural basis for many of the cognitive symptoms and behaviors reported by BC patients and survivors with CRF including impaired memory, difficulty concentrating, hypervigilance of internal body signals, and reduced engagement with the external environment (Anderson-Hanley et al., 2003).

### Conceptual Model of Neural Mechanisms of CRF in BC

The extensive functional changes that we report in the cortical and subcortical regions of the brain of BC patients and survivors allows us to propose a conceptual model that indicates global brain involvement in CRF (Figure 2).

Our model proposes CRF is associated with compensatory hyper activation of multiple cortical regions involved in memory and planning (dorsomedial pre-frontal cortex, medial frontal gyrus, inferior parietal cortex). Physical fatigue is associated with increased connectivity between the left inferior parietal lobule and superior frontal gyrus (region associated with memory and cognition). Mental fatigue is associated with increased connectivity of the DMN to the superior frontal gyrus. Increased connectivity of the insula (processes internal body signals) to the dorsolateral pre-frontal cortex may lead to greater self-referential appraisal of fatigue. Reduced connectivity between the salience network (anterior cingulate cortex) and the dorsal attention network (intraparietal sulcus) contribute to CRF through hypervigilance of internal bodily signals and reduced engagement with the external environment. Subcortical structures such as superior colliculus and pulvinar (thalamus) contribute indirectly to CRF by affecting sleep and alertness. Connectivity of the right precuneus to the superior colliculus (sleep regulation) and of the inferior parietal lobule to parahippocampal gyrus (memory encoding and retrieval) may be protective against the development of CRF. Each aspect of this conceptual model is discussed in detail below.

Functional changes in frontoparietal cortex are key contributors to CRF. CRF is associated with hyper activations of several regions in the frontoparietal cortex involved in memory and planning (dorsomedial pre-frontal cortex, medial frontal gyrus, inferior parietal cortex), (López Zunini et al., 2013; Menning et al., 2015, 2017). However, these hyper activations were not associated with any significant reduction in task performance suggesting that the observed hyper activations represent over-recruitment of brain regions and are a compensatory mechanism through which fatigued BC patients try to maintain adequate executive function. These findings are supported by a PET scan study in which BC patients showed reduced metabolism in the frontal regions in the resting state but hyper activations occurred when performing a memory recall task (Silverman et al., 2007). Though this study did not report PET scan findings in relationship to CRF, these findings suggest that task related hyper activations in breast CRF may represent a compensation for reduced brain metabolism at rest.

Connectivity changes between regions involved in processing memory and attention may explain the impaired memory and concentration commonly reported by BC patients with CRF. The superior frontal gyrus has previously been shown to be associated with poor memory and disrupted cognition in chronic fatigue patients (Lange et al., 2005). Increased connectivity between the left inferior parietal lobule and superior frontal gyrus may represent overuse of these regions in CRF (Hampson et al., 2015). Mental fatigue was associated with increased connectivity of the DMN to the superior frontal gyrus. The DMN is composed of a constellation of regions of the frontoparietal network and is involved with self-referential thought that occurs when a person is resting and not engaged with the external environment (Buckner and Vincent, 2007; Buckner et al., 2008). Increased connectivity of the DMN to the superior frontal gyrus likely increases self-referential thoughts leading to cogitation and a sensation of mental fatigue. Similarly, reduced connectivity between the salience network (anterior cingulate cortex) and the dorsal attention network (intraparietal sulcus) likely contribute to CRF through hypervigilance of internal bodily signals and reduced engagement with the external environment.

The insula, located deep to the lateral sulcus, has a key role in processing internal body signals (interoception) including sensation of fatigue. Prior studies have shown that connections of dorsolateral pre-frontal cortex to the insula provides “top down” regulation of mental and physical fatigue (Provencher, 1993; Beck et al., 2004). In BC survivors, CRF was associated with higher levels of the excitatory neurotransmitter glutamate in the insula as well as increased connectivity of the insula to the dorsolateral pre-frontal cortex. These findings suggest that CRF may occur due to greater self-referential appraisal of fatigue. Another subcortical structure widely implicated in fatigue in animal studies is the hippocampus such that the hippocampal involvement in fatigue correlates with disrupted memory consolidation (Harrington, 2012). One study in BC patients reported that CRF was associated with hyper activation of the hippocampus, likely as a compensatory effect to maintain memory (López Zunini et al., 2013).

Brain stem structures such as superior colliculus (involved in sleep regulation) indirectly contribute to CRF (Zhou et al., 2016). Connectivity between DMN and the superior colliculus was elevated in non-fatigued BC survivors as compared to those with fatigue suggesting that this connectivity pattern could be protective by inhibiting fatigue (Harris et al., 2017). Similarly, connectivity of the inferior parietal lobule to parahippocampal gyrus (memory encoding and retrieval) was higher in fatigued than non-fatigued survivors and may be protective against the development of CRF (Bohbot et al., 2015; Harris et al., 2017).

The above conceptual model of global brain involvement in BC patients is biologically plausible because CRF is a subjective complaint of widespread physical, cognitive, and emotional distress. This model is also compatible with the numerous immune/inflammatory, metabolic, neuroendocrine, and genetic biomarkers implicated in CRF (Saligan et al., 2015), which could potentially affect different regions and networks in the brain. In other conditions in which fatigue is a prominent symptom such as multiple sclerosis and Parkinson's disease, fatigue is associated with impaired global connectivity and reduced global efficiency of the brain (Sang et al., 2015; Vecchio et al., 2017). A conceptual model of global brain involvement in CRF suggests that graphical methods such as small-world network analysis that measure functional changes across the entire brain could be used to develop biomarkers to identify BC patients at high risk for developing CRF.

### Clinical Significance

We integrated the analysis of neuroimaging findings with a clinical outcome (fatigue score) to draw clinically meaningful conclusions that could advance the care of BC patients. First, frontoparietal hyper activations and reduced efficiency of the frontoparietal cortex was noted in BC patients at baseline even before the start of chemotherapy. Next, anxiety modulated the association between CRF and frontoparietal activations; however, fatigue was independently associated with functional brain changes after adjusting for the confounding effect of anxiety. Finally, functional changes in the frontoparietal network that supports executive function were a better predictor of post-treatment CRF than chemotherapy itself. Together, these findings indicate that BC patients, especially those scheduled to undergo chemotherapy, are at particularly high risk for developing post-treatment CRF. Furthermore, pre-treatment functional neural changes that are modulated by anxiety and fatigue are more important contributors to post-treatment CRF than chemotherapy. A neural biomarker that reliably predicts post-treatment CRF could further help to identify BC patients who would benefit from interventions to reduce anxiety and improve memory and concentration before initiation of chemotherapy.

The neural basis for CRF opens the door for developing treatments that modify these mechanisms. Two small studies included in this review have already demonstrated that interventions such as acupressure and mindfulness improve fatigue scores in BC survivors by modulating the connectivity between brain networks implicated in CRF. Larger studies will be required to determine if these treatments result in sustainable improvements in CRF in BC patients and survivors.

### Limitations

Our search process did not identify any PET scan and EEG studies that met our inclusion and exclusion criteria. We were unable to include two EEG studies that investigated CRF because majority of included subjects were male patients with solid tumors (lung, renal and gastrointestinal cancer) and only five patients had breast cancer (all male), (Jiang et al., 2019; Allexandre et al., 2020). Though not directly applicable to breast CRF, these studies provide useful information regarding central mechanisms underlying physical fatigue in patients with CRF. Specifically, these studies showed that physical fatigue in cancer patients is associated with altered electrical brain activity during an elbow flexor contraction. Allexandre et al. (2020) demonstrated that changes in theta (4–8 Hz) and beta (12–30 Hz) bands in the contralateral (to the fatigued limb) hemisphere were correlated with evoked muscle force, a marker of central fatigue. Jiang et al. (2019) used simultaneous EEG and EMG (electromyogram) recordings to measure functional coupling between brain and muscle signals during an elbow flexor contraction. As compared to healthy controls, CRF participants demonstrated significantly lower EEG-EMG coherence at the alpha (8~14 Hz) and beta (15~35 Hz) frequency bands indicating that physical fatigue in cancer patients may be related to impaired functional coupling between brain and muscle signals.

Relatively few studies have used PET scan to investigate functional brain changes in cancer patients or survivors. In a retrospective review of small cell lung cancer patients, Eshghi et al. (2018) reported regional differences (both increases and decreases) in (18)F-FDG uptake in the brain before and after cranial irradiation. Prior PET scan studies in BC patients have demonstrated increased activation when performing a memory task but reduced brain metabolism in the frontal regions in the resting state suggesting that increased activation represents a compensation for reduced metabolism at rest (Silverman et al., 2007). In a prospective study of 33 BC patients, Pomykala et al. (2013b) combined (18)F-FDG PET/CT scan with measurement of pro-inflammatory cytokine markers to demonstrate that metabolism in the medial pre-frontal cortex and anterior temporal cortex correlated with both memory complaints and cytokine marker levels in chemotherapy patients. Though these studies did not report on fatigue outcomes, these studies suggest that (18)F-FDG PET scan could provide greater insight into the effect of inflammatory and immune markers that cross the blood brain barrier on brain metabolism in CRF.

Included studies in our review were also of relatively small sample size, employed different study designs, neuroimaging methods, and were performed in a heterogeneous group of BC survivors or subjects in different stages of treatments. Additionally, studies used different cognitive tasks to explore a variety of brain regions and different cognitive processes are at play depending on the task at hand. Not all studies reported coordinates of regions activated which prevented us from performing a meta-analysis of brain regions associated with CRF. There is also considerable heterogeneity in the subject cohorts with respect to age, co-morbidities, and treatment status which affects our ability to distill precise neural functional changes in CRF. In future studies, a powerful approach to get around this issue of heterogeneity would be to perform a study with a repeated measure design, such as first neuroimaging fatigued individuals during treatment of their cancer, and then repeating imaging after successful treatment and when they are no longer fatigued.

### Future Directions

A prior systematic review has reported that immune/inflammatory, metabolic, neuroendocrine, and genetic biomarkers of CRF impact central nervous system function through their effect on brain neurotransmitters and neuropeptides (Saligan et al., 2015). Two small studies included in our review did not find a significant relationship between CRF, brain changes and peripheral biomarkers of stress and inflammation (Zick et al., 2014; Menning et al., 2015). Newer high resolution magnetic resonance spectroscopy techniques offer the opportunity to measure brain neurotransmitters such as glutamate with high precision and could provide greater insight into neural mechanisms of CRF (Marsman et al., 2017). High resolution neuroimaging techniques could also help to elucidate the role of subcortical structures such as hippocampus which have been shown to play an important role in fatigue in animal studies. Finally, a neural biomarker that reliably predicts patients at high risk for post-treatment CRF could be used to identify patients who would benefit from interventions for improving anxiety and memory before initiating chemotherapy. Such a neural biomarker could be developed using relatively easily available BOLD fMRI techniques and small-world network analysis.

Another important question is whether neural mechanisms identified in breast CRF can be applied to CRF with other cancers. Studies involving blood biomarkers indicate that immune, inflammatory, and genetic biomarkers of CRF vary with the type of cancer, stage of cancer, and type of treatment and could potentially exert differential effects on the central nervous system (Saligan et al., 2015). Large studies involving many different cancer types using standardized definitions of CRF and uniform neuroimaging protocols will be required to determine if an integrated conceptual model of CRF that extends across different cancer types can be developed.

In conclusion, cortical, subcortical and brain stem regions that process memory, interoception, internal and external attention are involved in neural mechanisms of CRF in BC patients and survivors. These findings not only provide a neural basis for many of the symptoms reported by BC patients with CRF, but they also provide useful directions to practicing clinicians for identifying high risk patients and to researchers for developing and testing neural biomarkers and treatments for CRF.

## Data Availability Statement

The original contributions presented in the study are included in the article/supplementary material, further inquiries can be directed to the corresponding author.

## Author Contributions

HR conceived the project. NA wrote the protocol, analyzed the data, and drafted the manuscript. AV and KZ conducted the search, the study selection, the data extraction, and risk of bias. All authors designed and approved the protocol, reviewed, and approved.

## Funding

This work was supported in part by NIH Grants R21-AG051981 and R01-MH107571.

## Conflict of Interest

The authors declare that the research was conducted in the absence of any commercial or financial relationships that could be construed as a potential conflict of interest.

## Publisher's Note

All claims expressed in this article are solely those of the authors and do not necessarily represent those of their affiliated organizations, or those of the publisher, the editors and the reviewers. Any product that may be evaluated in this article, or claim that may be made by its manufacturer, is not guaranteed or endorsed by the publisher.
